# Two fatal autochthonous cases of airport malaria, Belgium, 2020

**DOI:** 10.2807/1560-7917.ES.2022.27.16.2100724

**Published:** 2022-04-21

**Authors:** Wim Van Bortel, Bea Van den Poel, Greet Hermans, Marleen Vanden Driessche, Helmut Molzahn, Isra Deblauwe, Katrien De Wolf, Anna Schneider, Nick Van Hul, Ruth Müller, Leen Wilmaerts, Sophie Gombeer, Nathalie Smitz, Johanna Helena Kattenberg, Pieter Monsieurs, Anna Rosanas-Urgell, Marjan Van Esbroeck, Emmanuel Bottieau, Ula Maniewski-Kelner, Javiera Rebolledo

**Affiliations:** 1 Unit of Entomology, Institute of Tropical Medicine, Antwerp, Belgium; 2 Outbreak Research Team, Institute of Tropical Medicine, Antwerp, Belgium; 3 Clinical Laboratory, Jan Portaels General Hospital, Vilvoorde, Belgium; 4 Medical Intensive Care Unit, Department of General Internal Medicine, University Hospitals Leuven, Leuven, Belgium; 5 Department of Cellular and Molecular Medicine, KU Leuven, Leuven, Belgium; 6 Department of Laboratory Medicine, University Hospitals Leuven, Leuven, Belgium; 7 Intensive Care Unit, Jan Portaels General Hospital, Vilvoorde, Belgium; 8 Veterinary Service, Military Hospital Queen Astrid, Brussels, Belgium; 9 Royal Belgian Institute of Natural Sciences, Barcoding Facility for Organisms and Tissues of Policy Concern (BopCo), Brussels, Belgium; 10 Royal Museum for Central Africa, Barcoding Facility for Organisms and Tissues of Policy Concern (BopCo), Tervuren, Belgium; 11 Unit of Malariology, Department of Biomedical Sciences, Institute of Tropical Medicine, Antwerp, Belgium; 12 Department of Clinical Sciences, Institute of Tropical Medicine, Antwerp, Belgium; 13 Department of epidemiology and infectious diseases, Sciensano, Brussels, Belgium

**Keywords:** malaria, autochthonous transmission, Belgium, airport malaria, Odyssean malaria, *Plasmodium falciparum*

## Abstract

We report an outbreak investigation of two fatal cases of autochthonous *Plasmodium falciparum* malaria that occurred in Belgium in September 2020. Various hypotheses of the potential source of infection were investigated. The most likely route of transmission was through an infectious exotic *Anopheles* mosquito that was imported via the international airport of Brussels or the military airport Melsbroek and infected the cases who lived at 5 km from the airports. Based on genomic analysis of the parasites collected from the two cases, the most likely origin of the *Plasmodium* was Gabon or Cameroon. Further, the parasites collected from the two Belgian patients were identical by descent, which supports the assumption that the two infections originated from the bite of the same mosquito, during interrupted feeding. Although airport malaria remains a rare event, it has significant implications, particularly for the patient, as delayed or missed diagnosis of the cause of illness often results in complications and mortality. Therefore, to prevent such severe or fatal outcomes, we suggest a number of public health actions including increased awareness among health practitioners, especially those working in the vicinity of airports, and increased surveillance of exotic mosquito species at airports.

## Background

Malaria is a protozoan disease caused by the *Plasmodium* parasite and transmitted by the bite of an infectious *Anopheles* mosquito. In Belgium, malaria is a travel-associated infection. Between 2016 and 2019, 327 to 420 cases were reported annually by the Belgian National Reference Laboratory. In connection with the coronavirus disease (COVID-19) pandemic and the subsequent decrease in travel, the number of cases dropped to 179 in 2020. Local transmission is only sporadically reported in Belgium. In 1995, a cluster of six cases of airport malaria, including five cases with *Plasmodium falciparum* and one case with *Plasmodium ovale*, was reported from the international airport of Brussels, involving three airport employees and three visitors who had not travelled abroad; one patient died [1]. In 1997, a possible case of port malaria was described in Ghent [2] and in 1998, a case of airport malaria was reported from the regional airport of Oostende [3]. In 2008, another case was notified in Brussels, labelled as suitcase malaria [4]. In January 2015, a women in her 70s was diagnosed with *P. falciparum* malaria near Antwerp. The exact route of transmission has not been elucidated, but suitcase malaria was suspected [5].

The reporting of suspected autochthonous malaria is mandatory in Belgium. In case of suspected local transmission, different hypotheses of the potential source of infection need to be investigated [6]. Firstly, travel-related malaria, acquired in endemic zones, needs to be considered and excluded. Secondly, the investigation should assess the possibility of induced malaria, i.e. malaria not acquired from a mosquito but for example through blood transfusion or nosocomial transmission. Thirdly, introduced malaria, i.e. the possibility of transmission by a local *Anopheles* species needs to be examined which infected itself by taking a blood meal on a gametocyte carrier who got the infection in an endemic area. Finally, Odyssean malaria, which results from the bite of an imported infectious exotic *Anopheles* mosquito, can be considered [7].

### Outbreak detection

On 29 September 2020, during the second COVID-19 wave in Belgium, a person in their 80s with obesity and arterial hypertension presented to the emergency ward of a peripheral hospital with acute-onset diarrhoea and dyspnoea for 1 day. The person had fever (38.7 °C) at the time of admission and was conscious and oriented. Laboratory investigation showed elevated inflammatory markers, metabolic acidosis, and liver and kidney failure (Table 1, Case 1). The person tested negative for severe acute respiratory syndrome coronavirus 2 (SARS-CoV-2). Blood cell count showed a thrombocytopenia of 88 × 10^9^/L, but no anaemia. Despite antibiotic therapy and supportive treatment, the patient rapidly deteriorated and died of refractory shock on 30 September. Blood cultures sampled at admission remained negative for bacterial growth. Diagnosis of severe malaria caused by *P. falciparum* was made retrospectively (parasitaemia at admission of 8% of the red blood cells) when malaria was diagnosed in the second case.

**Table 1:** Laboratory values at admission, fatal autochthonous malaria cases, Belgium, 2020 (n = 2)

	Reference range	Case 1	Case 2
Haemoglobin, g/dL	11.9-14.6	15.7	15.8
Red blood cells, 10^6^/µL	3.9-5.1	5.6	5
White blood cells, 10^3^/µL	4.5-12.7	8.6	13
Platelets , 10^9^/L	173-390	88	24
C-reactive protein mg/L	0-5	117.5	335
Blood glucose, mg/dL	70-100	243	97
Sodium, mmol/L	136-145	125	132
Potassium, mmol/L	3.4-4.5	4.6	4.2
Chloride, mmol/L	98-107	90	92
Bicarbonate, mmol/L	21-28	17.5	19.5
Lactate, mmol/L	0.2-2.2	4.7	7.1
Alanine aminotransferase, U/L	<30	44	61
Aspartate aminotransferase, U/L	<33	99	168
Gamma-glutamyltransferase, U/L	<40	42	70
Alkaline phosphatase, U/L	35-105	129	66
Lactic dehydrogenase, U/L	240-480	1,178	1,622
Urea, mg/dL	17-49	51	95
Creatinine, mg/dL	0.5-0.9	1.01	1.51

The second case was a person in their 80s, the spouse of the Case 1. The person was admitted on 30 September 2020 to the same hospital with a 1-week history of cough, dyspnoea and fever. Upon hospital admission, the patient was conscious with a temperature of 38.2 °C. The person tested negative for SARS-CoV-2. The patient was tachycardic (120/min), tachypnoeic (27/min) and required supplemental oxygen (9 L). Broad-spectrum antibiotic therapy was initiated. Laboratory investigation showed elevated C-reactive protein (up to 335 mg/L), neutrophilic leukocytosis with myeloid precursor cells and severe thrombocytopenia (24 × 10^9^/L) but no anaemia. Metabolic acidosis with lactate levels of 7.1 mmol/L and haemoglobinuria were present, as well as elevated D-dimers and lactate dehydrogenase (Table 1). Because of haematological abnormalities, the blood sample was ‘flagged’ by the analyser (XT4000i, Sysmex, Norderstedt, Germany). Microscopic investigation by the laboratory technician revealed *P. falciparum* trophozoites with a parasitaemia of 30% of red blood cells. A diagnosis of severe malaria was made, based on the dyspnoea, hyper-parasitaemia and lactic acidosis [8]. Since artesunate was not available in the hospital, quinine was immediately administered intravenously as well as supportive intensive care to stabilise the patient. A transfer to a tertiary hospital was organised within 5 h and intravenous artesunate was immediately started during transport. In the intensive care unit, the patient rapidly developed respiratory failure, shock and acute kidney failure. Despite pharmacological haemodynamic support, continuous renal replacement therapy, correction of hypoglycaemia, metabolic acidosis further progressed and rhabdomyolysis developed. The patient died within 48 h after transfer from refractory shock and multiple organ failure.

Samples from both patients were sent to the malaria national reference laboratory at the Institute of Tropical Medicine Antwerp (ITM) for confirmation. Parasite density quantification was assessed according to the World Health Organization's standards for microscopy [9]. Microscopy confirmed *P. falciparum* trophozoites at a parasite density of 407,144 asexual parasites (AP)/µL (8.16%) for the first case and 1,170,852 AP/µL (29.20%) for the second case. The presence of *P. falciparum* was further confirmed with PCR using the method described by Cnops et al., with quantification cycle (Cq) values of 12.88 and 12.32, respectively [10].

As neither cases had travelled recently, the cases were reported to the regional health authorities on 1 October 2020 as part of the mandatory notification when autochthonous malaria is suspected. It also triggered an outbreak investigation to assess the possible route(s) of transmission in order to put prevention and control measures in place if needed. Here, we report the outbreak investigation of the two fatal cases of autochthonous *P. falciparum* malaria that occurred in the vicinity of the international airport Brussels and the military airport of Melsbroek in September 2020.

## Methods

### Outbreak investigation

We investigated various hypotheses of the potential source of infection. We carried out an epidemiological investigation around the cases in order to assess the hypothesis of travel-related malaria (hypothesis 1) and of induced malaria (hypothesis 2). We performed an entomological investigation in and around the house of the cases in search for exotic and native *Anopheles* mosquitoes. We searched for possible imported malaria cases (subsequently called index cases in accordance with the WHO malaria terminology [11]) near the place of residence of the cases to assess the possibility of introduced malaria (hypothesis 3). Further, the hypothesis of Odyssean malaria (hypothesis 4) was assessed. In order to advance the investigation, the parasite genomes of the two samples collected from the cases were analysed by whole genome sequencing (WGS) to assess the most likely origin of the parasite.

### Epidemiological investigation

We carefully assessed the travel history of the cases by asking their family members about past travels of the cases. The transmission through blood transfusion or nosocomial transmission was assessed at the hospital where the patients were admitted. Moreover, we investigated the national surveillance data on malaria cases: postal codes from cases with imported *P. falciparum* infections in the months of August and September 2020 were compared to the postal code of the cases in order to identify a possible index case (see section 'Flight information' for the rationale behind the considered time period).

### Entomological investigation

The entomological investigation searched for the presence of *Anopheles* mosquitoes in the house and garden of the cases, and in a wider area of 500 m around the house depending on the existence of potential larval habitats.

On 9 October, two entomologists inspected the house of the cases for the presence of adult mosquitoes using a mouth aspirator and a torch. On the same day, they conducted a search for larvae in the garden of the house and set up a BG Sentinel trap (Biogents, Regensburg, Germany) baited with BG Lure and CO_2_. The trap's content was collected 1 week later. In addition, a mosquito collected inside the house in the week after the cases' death by one of the family members was also taken for species identification. In case the collected mosquitoes in the house belonged to the *Anopheles* genus, the head and thorax were to be tested for *Plasmodium* spp. using the PCR method described in by Canier et al. [12].

On 12 October a mosquito larval collection was organised in a 500 m radius around the house of the cases. Different potential larval habitats were inspected including tree holes, ponds, sewers and any water-holding containers that were present in the gardens of the neighbours and in the surroundings. Outside the 500 m radius, in a nearby nature reserve, the marsh was inspected, and in the nearby forest, tree holes and a pond were checked.

On 13 October, two members of the Veterinary Service of Belgian Defence inspected the outside area of the military airport of Melsbroek to check for larval habitats.

Adult mosquitoes and larvae were morphologically identified to genus level. Specimens of the *Anopheles* genus were further identified to species level [13,14]. DNA barcoding confirmed the morphological species identification [15].

### Genomic analysis of parasite origin

We extracted DNA from the blood samples of the two cases and generated WGS reads on the NovaSeq platform (Illumina, San Diego, US). The sequencing data are available on the NCBI Short Read archive under accession numbers SRR16601879 and SRR16601880. The analysis used the *P. falciparum* Community Project Pf6 WGS database in addition to WGS data from Gabon for comparison (for details on the procedures used to generate and combine the sequencing data, see the Supplement) [16,17]. The majority of the samples in these databases were collected between 2007 and 2015 and only from few sites in a limited number of countries. The isolates from Gabon were collected in 2012. The geographical origin of the two *P. falciparum* isolates detected in Belgium was explored using a principal component analysis (PCA) as implemented in the scikit-allele python module and using a discriminant analysis of principal components (DAPC) from the adegenet package in R [18]. The first analysis did not use a priori knowledge on the possible origin of the samples; the second calculated the discriminant components using country information (see the Supplement for the figure displaying the PCA and DAPC results, as well as details on the applied methodology).

The relatedness between the two Belgian isolates and samples nearest in the PCA was subjected to identity-by-descent (IBD) analysis, which indicates common ancestry between strains, using the R package isoRelate [19]. Nucleotide sequences are IBD if they are identical and inherited without recombination. isoRelate performs pairwise relatedness mapping on genomes from haploid isolates using a first order continuous time hidden Markov model allowing for multiple infections as often observed in *Plasmodium* infections. Pairwise comparisons between isolates from Belgium and countries in the identified cluster (Benin, Cameroon, Democratic Republic of Congo (DRC), Gabon and Nigeria) were conducted using the same package, and the proportion of the genome sharing IBD was calculated.

### Flight information

Based on the onset of symptoms of the cases, the most likely period of transmission was before 15 September. In case of induced transmission, the index case should have arrived in Belgium in the first half of August to allow the development of the parasite in a local *Anopheles* mosquito. Therefore, we evaluated the flight volumes over the period 1 August to 15 September 2020 and compared this with the same period in 2019. We obtained data directly from the international airport Brussels and the military airport of Melsbroek.

## Results

### Epidemiological and clinical case investigation

Neither of the cases had travelled outside Belgium for more than 50 years. The cases spent most of their time inside their home and only went to nearby shops. They did not get any blood or organ transfusion in the last 5 years and were not admitted to any hospital wards 3 years before the malaria episode, excluding transmission through substances of human origin and nosocomial transmission. In total, 15 imported *P. falciparum* infections were reported in Belgium in August and September 2020. None of these cases lived close to the cases and could have been the source for infection of a local *Anopheles* mosquito. However, the two cases were living 5 km from the international Airport Brussels and the military airport Melsbroek, therefore we suspected Odyssean malaria (Table 2).

**Table 2:** Outbreak investigations of fatal autochthonous malaria cases, Belgium, 2020 (n = 2)

Hypothesis	Case investigation	Conclusion
1. Travel-related malaria, acquired in endemic zones	No recent travel history: none of the patients had travelled to an endemic area for more than 50 years. No travel of another member of the family to an endemic area nor visitors from abroad. No relapsing malaria as the causative pathogen of these cases was *Plasmodium falciparum*.	Can be excluded
2. Induced or not mosquito-transmitted malaria	No blood transfusion or organ transplantation in the last 5 years. Nosocomial transmission could be excluded, as the cases were not admitted to any hospital wards in the last 3 years.	Very unlikely
3. Introduced malaria	Based on postal codes of patients with imported *P. falciparum* infections in August and September 2020 (n=15), no travel-related cases could be identified that could have been the source of infection of a native vector species. *Anopheles claviger* and *Anopheles daciae* were found inside the house and in a 500 m buffer zone around the house, respectively; these species are not known as vectors of *P. falciparum*.	Very unlikely
4. Odyssean malaria or acquired through imported infectious exotic mosquito	The cases lived at 5 km from the international airport Brussels and the military airport Melsbroek. An imported exotic *Anopheles* infected could have flown from one of these places to the cases' house.	Most likely explanation

### Entomological investigation

In the house of the cases, a single dead female mosquito was collected on the windowsill of the garage. The mosquito was identified as *Anopheles claviger* using morphology and DNA barcoding (GenBank accession number: MZ490542). The thorax and head portion of the female mosquito specimen was tested for the presence of the malaria parasite (*Plasmodium* spp.) and was negative. The mosquito collected by a family member was identified as *Culex pipiens/Cx. torrentium.* Using a mouth aspirator, one adult male of the native mosquito *Cx. pipiens/Cx. torrentium* was collected from a rain barrel in the garden. The BG-Sentinel trap collected an additional 10 specimens of *Cx. pipiens/Cx. torrentium*. In the garden, a total of 37 containers of three different types of potential larval habitats were inspected, more specifically 30 plastic containers (such as flower pots, buckets and rain barrels), five plastic sheeting and two metal containers (e.g. drinking troughs). Most plastic containers and tarpaulins were positive for the presence of *Cx. pipiens/Cx. torrentium*. No other mosquito species were found in the vicinity of the house.

In the area around the cases' house, 23 gardens were visited and a total of 139 containers of 11 different types of potential larval habitats were inspected. In the gardens and the nearby forest, only *Cx. pipiens/Cx. torrentium* larvae and pupae were collected. In the nature reserve, one pupa of the native mosquito *Anopheles daciae* was collected. Its identification was based on morphology and DNA barcoding (GenBank accession numbers: COI MZ490543 and ITS2 MZ490544). Also the native *Cx. pipiens/Cx. torrentium* and *Culiseta annulata* were collected in the marsh of this nature reserve.

On the site of the military airport Melsbroek, no potential larval habitats for mosquito were found and no mosquitoes were collected.

### Genomic analysis of parasite origin

**Figure 1:**
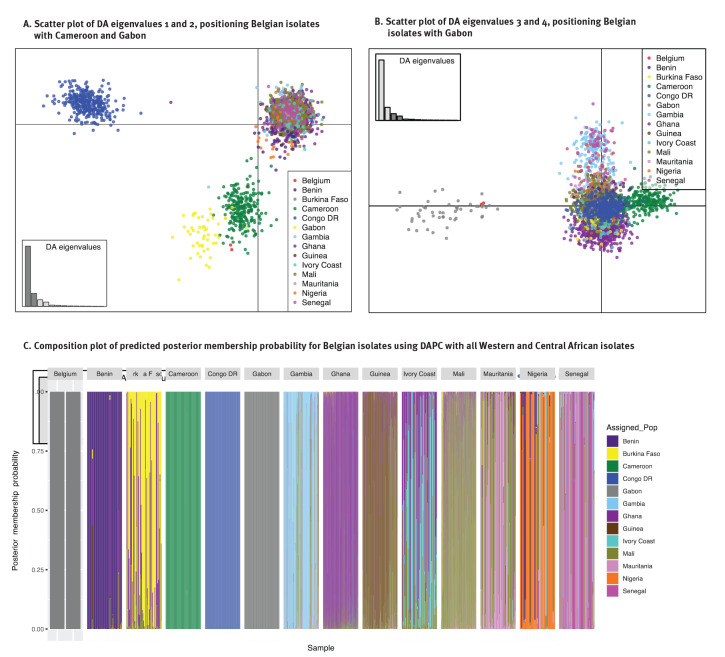
Discriminant analysis of principle components, autochthonous malaria cases, Belgium, 2020 (n = 2)

DA: discriminant analysis; DAPC: discriminant analysis of principle components.

When compared with a database of *P. falciparum* genome sequences from across the world, the two Belgium isolates were placed in the large cluster with isolates from Africa and South America in the PCA, based on the genetic variation observed in those samples (Supplementary Figure 1A shows the PCA figure of the first four principal components). In the PCA on a subset of the samples from Africa and South America (Supplementary Figure 1B), the Belgian isolates were placed near West and Central African strains and closest to isolates from countries at the border between West and Central Africa, but not the DRC (samples collected in Kinshasa). By enhancing the between-country variation using DAPC, the Belgian isolates were positioned closest to Gabon and Cameroon, and both isolates were predicted with the highest posterior probability (0.99) to belong to the cluster with samples from Gabon in the DAPC including samples from West and Central Africa (Figure 1).

The parasite isolates collected in Belgium were 100% IBD, indicating that they were inherited from the same ancestor. However, little IBD was observed between the Belgian isolates and the selected samples from countries in West and Central Africa, indicating no recent shared ancestry. The exception was one isolate from Gabon from 2012 showing 2.9% IBD with the Belgian isolates. This low IBD indicates a shared, although distant ancestry. The proportion of the genome in IBD in the pairwise comparisons within countries was 2.9% (range: 0.3–100) and larger than generally found in pairwise comparisons of isolates from different countries (1.8%; range: 0.68–8.0).

### Flight information

**Figure 2:**
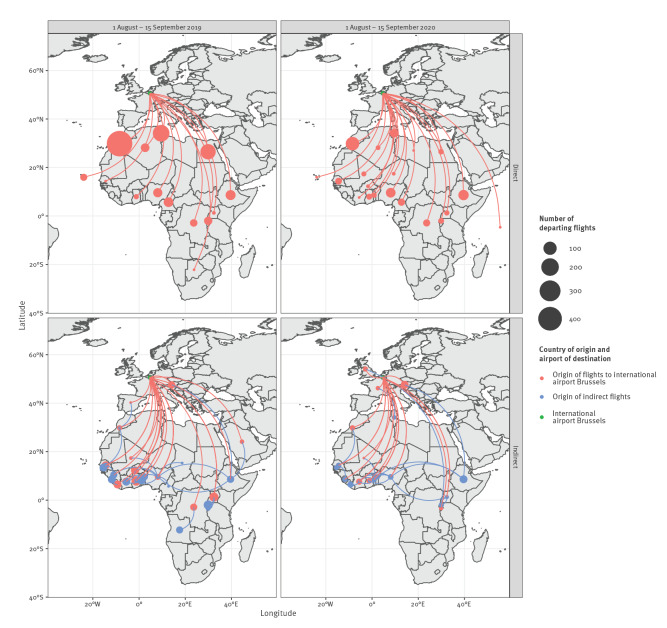
Direct and indirect flights from Africa to the international airport Brussels, 1 August–15 September 2019 and 2020

Over the period 1 August to 15 September 2020, 471 flights – cargo and passengers flight combined – arrived from Africa at the international airport Brussels. Of them, 154 were direct flights originating from West Africa, 20 from Cameroon and none from Gabon. Compared with 2019, 65% fewer flights arrived from Africa at the international airport Brussels in 2020. Despite this decrease, while direct flights had arrived from 14 African countries at the international airport Brussels in the period 1 August to 15 September 2019, 20 African countries were connected via direct flight to the international airport Brussels in the same period in 2020 (Figure 2). Most of the indirect flights had a stop-over in an African country (Figure 2). In the same period, 1 August to 15 September 2020, six direct and six indirect flights arrived from West Africa at the military airport Melsbroek, but no flights arrived from Gabon or Cameroon.

## Outbreak control measures

As this transmission event was an isolated occurrence, the outbreak control measures included primarily communication to the population living in the surroundings of the cases. This included a press conference organised by the commune and information leaflets distributed to the population of the commune. Further, health professionals of the Region Flanders were informed about the cases to increase awareness. The outbreak investigation and the implementation of control measures are the responsibility of the regional health authorities. At national level, a risk assessment was made by the Risk Assessment Group coordinated by Sciensano (The federal Public and Veterinary Health Institute) and approved by the Risk Management Group. No further control measures were deemed necessary.

## Discussion

We described two fatal cases of autochthonous *P. falciparum* malaria in Belgium in 2020, at the beginning of the second COVID-19 wave in Belgium. Based on the information collected during the outbreak investigation the most likely route of transmission was through an infectious exotic *Anopheles* mosquito that arrived via the international airport of Brussels or the Military airport Melsbroek and infected the cases.

The entomological investigation in and around the cases' house revealed the presence of two native *Anopheles* species, *An. claviger* and *An. daciae*. The former was a vector species of *Plasmodium vivax* in the eastern Mediterranean region [20]. The vector competence of *An. daciae* is not known as this species has only recently been described [21] but the species could possibly have been responsible for *P. vivax* transmission that was attributed to *An. messeae* [20,22]. Neither species is known as vector of *P. falciparum*. During the entomological investigation, we did not find *Anopheles plumbeus*, a competent *P. falciparum* vector [23] which was incriminated as vector in two autochthonous *P. falciparum* cases in Germany in 1997 [24]. Furthermore, the epidemiological investigation of imported malaria cases in the vicinity of the cases did not reveal any travel-associated malaria case that could have been identified as index case. Combining these observations, it is very unlikely that local *Anopheles* mosquitoes were responsible for the two local malaria cases.

One of the cases developed symptoms around 22 September. Given the incubation period of 8–12 days for *P. falciparum,* the transmission most probably took place in the second week of September. During that period, Belgium experienced high temperatures [25], similarly as in 1995 when a cluster of six airport malaria cases were reported from the international airport of Brussels. Because of the COVID-19 pandemic, fewer flights arrived from the likely location of origin of the parasite in 2020 compared with 2019, which is likely to have reduced the probability of entry of exotic mosquitoes through this import route. However, the hot weather could have favoured the survival of introduced exotic *Anopheles* mosquitoes. As the cases lived 5 km from the international airport Brussels and the military airport Melsbroek, an infectious exotic *Anopheles* mosquito could have survived and reached their house. In fact, the import of exotic *Anopheles* species does occur in Belgium. In 2017, a female *Anopheles pharoensis*, a malaria vector from Africa, was caught at the cargo airport of Liège during the Monitoring of Exotic Mosquito species Project [26,27]. Also in the Netherlands, exotic *Anopheles* species are sporadically caught at airports [26]. The isolates from the two Belgian patients were identical by descent, which supports the assumption that the two infections originated from the same exotic mosquito, via interrupted feeding.

Odyssean malaria, also called airport, port or suitcase malaria, results from the bite of an imported infectious exotic mosquito [7]. The conclusion of this possible route of infection is often based on excluding other hypothesis, since a direct observation of this event is very unlikely. Yet, the advanced genetic analysis provided additional elements to our investigation. The genetic signatures of the isolates from the cases indicated that they were most closely related to isolates from Gabon. Parasite subpopulation structure was observed in the dataset separating West, Central and South-Central African populations, and the Belgian strains clustered with the central region of Africa near Gabon and Cameroon. One sample from Gabon showed a low IBD (2.9%) with the Belgian isolates, suggesting a shared, although distant ancestry. However, in that part of Africa, there is high transmission and an intensely recombining parasite population, therefore it is expected that IBD proportions shared between isolates within the same country are not very high, and decrease over time [17]. Predicting the country of origin from genomic data largely depends on the completeness of the parasite WGS database. In this context, major limitations existed in the available data in terms of (i) geographical representation – the more countries and sites within a country are represented in the genomic dataset, the higher the accuracy of predicting the origin – and (ii) the time difference between the collection of the reference dataset and the collection of isolates for prediction, as parasite population structure changes over time because of frequent recombination. In our analyses, we used the data available at online sources. Larger datasets of more recent isolates collected from multiple countries and areas within countries are required to predict more precisely the origin of imported isolates. Networks of collaborating research institutes and/or reference laboratories in endemic and non-endemic areas involved in isolate collection and/or malaria genomics and surveillance, as well as data sharing policies, could contribute towards expanding the existing datasets.

In the period 1987 to 1995, 31 cases of airport malaria were reported in Europe [28], including the cluster of six cases in Belgium in 1995 [1]. More recently, in 2019, two airport-associated malaria cases were reported in Germany [29]. In 2020, besides the two cases in Belgium, three airport malaria cases were reported in France [30]. Despite these occurrences, Odyssean malaria remains a rare event. Yet, it has significant implications, particularly for the patient, as delayed or missed diagnosis of the cause of illness often results in high rates of complications and mortality. This is particularly the case for elderly people, because even for severe disease, initial symptoms can be mild, and a subjective feeling of fever can go unnoticed by the patient when confusion arises in conjunction with high body temperature. Moreover, physicians may not consider malaria as differential diagnosis because of missing travel history and interpret symptoms and laboratory findings as associated with underlying diseases.

## Conclusion and public health messages

The occurrence of the two fatal autochthonous malaria cases in Belgium indicates a number of public health actions that should be considered. A good human surveillance is essential to understand the epidemiology of travel-related malaria, and a timely notification to local health authorities whenever there is a suspicion of local malaria is of paramount importance to rapidly put an outbreak investigation in place. Raising awareness among health care practitioners working near airports is essential as they should consider malaria as a differential diagnosis when laboratory and/or clinical features such as recurrent and unexplained fever or severe thrombocytopenia are observed even without obvious exposure abroad. In such a case, laboratories could consider systematic microscopic examination of blood or even rapid malaria antigen tests, particularly in the presence of unclear thrombocytopenia or haemolysis. In addition, artesunate should be continuously positioned, in particular in hospitals in the vicinity of international airports. Further, our observations point to the need for vigilance at airports for import of exotic mosquito species, the necessity for continuous monitoring in and around airports as well as the need for appropriate control of mosquitoes at airports, including the luggage and cargo spaces, and in airplanes, and this particularly during the mosquito season from May till October.

## Supplementary Data

554000 Supplement
